# The Influence of Background Materials on the Radiative Cooling Performance of Semi-Transparent and Opaque Textiles: A Theoretical and Experimental Analysis

**DOI:** 10.3390/polym16162264

**Published:** 2024-08-09

**Authors:** Lea Zimmermann, Ablimit Aili, Thomas Stegmaier, Cigdem Kaya, Götz T. Gresser

**Affiliations:** 1 German Institutes of Textile and Fiber Research (DITF), Koerschtalstrasse 26, 73770 Denkendorf, Germany; 2 College of Engineering, Nanyang Technological University, Singapore 639798, Singapore; 3 Institute for Textile and Fiber Technologies (ITFT), University of Stuttgart, Pfaffenwaldring 9, 70569 Stuttgart, Germany

**Keywords:** radiative cooling, calculation of cooling power, comparison of semi-transparent and opaque textile materials

## Abstract

This paper investigates the theoretical and experimental cooling performance of textile materials utilizing radiative cooling technology. By applying Kirchhoff’s law, the emissivity of surfaces is determined, revealing that materials with high transmission values can achieve comparable cooling performance to those with high reflection values. Notably, materials exhibiting moderate reflectance and transmittance in the solar range tend to absorb minimal solar radiation, thus offering high theoretical cooling performance. However, practical applications like building envelopes or clothing present challenges due to the impact of background radiation on overall cooling capacity. Despite their intrinsic cooling properties, a significant portion of solar radiation is transmitted, complicating matters as the background can significantly affect overall cooling performance. This study provides a solution that accounts for the influence of background materials. Based on spectral data, various background materials and their impact on different semi-transparent comparison materials can be considered, and cooling performance can be simulated. This enables the simulation of cooling performance for various application scenarios and facilitates comparisons between transparent, semi-transparent, and opaque textile materials.

## 1. Introduction

Technologies such as radiative cooling offer a sustainable and energy-free solution by using the wavelength ranges of the atmosphere that are transparent to electromagnetic radiation, the so-called atmospheric window (8–13 µm), to emit thermal radiation into the colder (3 K) outer space [1]. 

Textile materials have gained importance in this field due to their versatile applications in areas such as body heat management and technical applications for membrane roofs, facade elements, awnings, or car covers. Other possible uses include glacier protection. 

The diverse application possibilities mean that textile materials are encountered and applied to various substrates, whether it be glacier ice, car paint surfaces, roofs, house walls, or even human skin. At the same time, various textile materials are developed for these different application scenarios, and their cooling properties are investigated.

Previous publications on textile systems have been focused on personal thermal management, particularly in the context of clothing. The human body dissipates heat to the immediate surroundings through three different pathways: evaporation cooling, convection, and radiative cooling. Radiative cooling accounts for approximately 60% of the three transmission pathways [2]. Developments for direct body cooling focusing on thermally transparent materials [3,4,5], utilizing the body as an emitting component. The skin itself is considered a very good emitter in the mid-infrared (MIR) range, allowing a large portion of body heat to be directly emitted to the environment through the skin surface [6]. 

To measure the cooling capacity and temperature reduction, silicone is mostly used to simulate the skin as an underlying background material [3,4,5]. 

Conversely, in the field of technical textiles, the objective is to shield from solar heat and reflect the sunlight irradiation for applications like tents or buildings during the day while selectively emitting heat within the atmospheric window, thereby creating an energy-free cooling effect. To achieve desired radiative-optical properties for cooling, publications in the field of technical textiles have focused on specific fiber structures [7,8,9,10] and textile substrates [11,12], as well as multilayer constructions [10,13].

Commonly, comparisons between coated and uncoated textile materials are employed to evaluate the advantage of cooling materials. Different measurement setups are used in the literature to measure temperature differences and cooling power compared to untreated textiles. Copper or aluminum metal plates are mostly used as the underlying background material [8,9,10,11,12,13]. 

In most of the research papers, the cooling capacity of textile materials is compared and validated experimentally under specific surrounding conditions. To calculate the theoretical cooling performance and validate the cooling power compared to untreated textiles, further challenges arise. 

The partial transparency of uncoated textiles can significantly influence heat transfer and temperature measurements, depending on the substrate used. Additionally, while textiles with higher solar reflectivity are expected to perform better, those with lower solar reflectivity and higher solar transmissivity may also exhibit high theoretical cooling power due to their lower overall absorption in the solar spectrum. 

**Figure 1:**
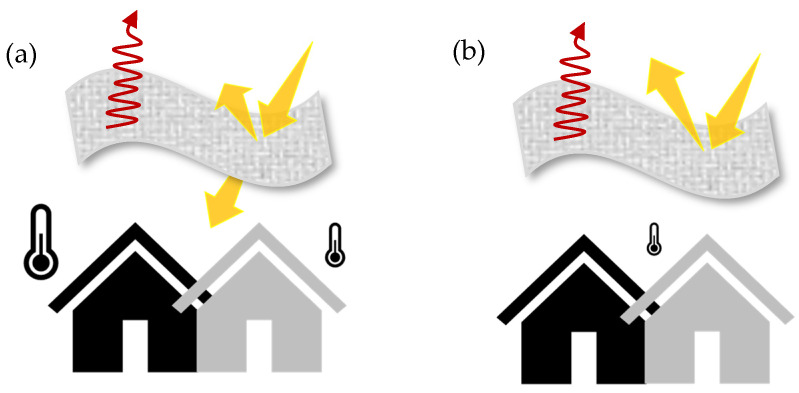
Schematic representation of the differences between semi-transparent and opaque textile materials. (**a**) Solar radiation is transmitted through a partially transparent textile during the day. The building underneath is heated to different degrees depending on the substrate and background material. (**b**) Solar radiation cannot transmit through opaque samples, so the cooling capacity is independent of the substrate and background material, and the temperature below stays constant.

In the application context of, e.g., building envelopes or tents, however, this does not lead to any significant temperature reduction (see Figure 1). Despite their intrinsic cooling properties, a significant portion of solar radiation is transmitted. In practical terms, this means that the underlying background might experience heating effects even if the materials themselves remain cool, posing a challenge in achieving optimal cooling outcomes in certain applications during the daytime. 

The cooling capacity $q_{cooling}$ in W/m^2^ is represented in a net radiation balance (1) as follows [14,15]:

(1) $$q_{cooling}=q_{rad}-q_{solar}-q_{atm}-q_{nonrad}$$

Here, $q_{rad}$ describes the thermal infrared radiation of the radiative cooling material, $q_{solar}$ represents the absorption of solar radiation, $q_{atm}$ denotes the absorption of downward atmospheric thermal radiation, and $q_{nonrad}$ accounts for the absorption of heat through convection and conduction.

The emission of the surfaces at a temperature T can be calculated by integrating thermal emission over all wavelengths and directions [14]:

(2) $$q_{rad}=\int cos\int_{0}^{\infty }\varepsilon_{r}\left(\lambda ,\Omega \right)I_{bb}\left(\lambda ,T_{r}\right)d\lambda$$

Ω describes the angle between the direction of radiation and the normal to the surface, $\varepsilon_{r}\left(\lambda ,\Omega \right)$ is the emissivity of the surface depending on wavelength and direction, and $I_{\mathrm{BB}}$ describes the blackbody radiation depending on wavelength and temperature.

The emissivity of the surface $\left(\varepsilon_{r}\right)$ is the variable that can be influenced by material physiological changes and measured and calculated using spectral measurements.

Based on the spectral analysis, the spectral absorption A(λ) is calculated from the spectral transmission T(λ) and the spectral reflection R(λ) by R(λ) + T(λ) + A(λ) = 1. The reflectance and the transmittance are measured using spectral measurement devices [7,8,9]. The emissivity of the surface $\varepsilon_{r}$ can be determined based on Kirchhoff’s law by $\varepsilon_{r}$(λ) = A(λ) = 1 − R(λ) − T(λ). The focus is solely on the material itself, adhering to Kirchhoff’s law and E = 1 − T − R. The result is that materials with both moderate reflectance and transmittance in the solar range absorb minimal solar radiation, resulting in high theoretical cooling performance. 

For opaque samples, T(λ) = 0 so that $\varepsilon_{r}$(λ) can be calculated by $\varepsilon_{r}$(λ) = 1 − R(λ) [16,17].

Given the importance of considering underlying background materials for both real applications and test setups, different background materials can significantly impact cooling power. This study aims to address the challenge of calculating the cooling power of semi-transparent and opaque textile materials by proposing a methodology that considers the influence of background materials. Leveraging spectral data, various background materials and their impact on different semi-transparent materials can be evaluated, facilitating the simulation of cooling performance across diverse application scenarios. We also tested and evaluated the influence of different background materials experimentally to demonstrate the impact based on the transparency of textile materials.

This approach enables comparisons between transparent, semi-transparent, and opaque materials under standardized conditions, thereby providing a comprehensive understanding of the cooling capabilities, especially for textile applications. 

## 2. Materials and Methods

### 2.1. Textile Substrate Materials 

As a textile sample, a standard polyester fabric (UTT, Krumbach, Germany) with a weight per area of 65 g/m^2^ was utilized, as well as a standard polyamide fabric (UTT, Germany) with a weight per area of 150 g/m^2^. The textile samples serve as the uncoated reference samples, possessing moderate solar transmissivity and reflectivity (details of the spectral curve can be extracted from the Supplementary Materials Figure S1). These textile materials were selected based on the type of material and the area weight. Polyester and polyamide are among the materials that, alongside ETFE, are typically used in membrane and tent construction. For the area weight, a relatively very light fabric (65 g/m^2^) and a significantly heavier fabric (150 g/m^2^) were chosen to represent the wide range of higher and lower solar transmissivity materials. The polyester sample (PES 65 g/m^2^) is used additionally as the substrate material for the cooling coating, resulting in an opaque cooling material for comparison.

### 2.2. Background Materials

A standard aluminum foil (15 µm) and a standard black foil (30 µm) are utilized as the background materials. Aluminum offers high solar reflectivity and low MIR emissivity, whereas the black material provides low solar reflectivity and high MIR emissivity, allowing representation of both extremes. Additionally, a metal plate is used as a background material, which is commonly employed in the experimental setup. This consists of a standard aluminum metal plate with a thickness of 0.5 mm. The spectral data are added in Supplementary Materials Figure S2. 

### 2.3. Coating Formulation for the Opaque Cooling Textile Material

For the coating formulation of the cooling textile material, silicone (LR6250F, Wacker Chemie AG, München, Germany) is used as the matrix material. Silicone is a material that has already been used for radiative cooling applications in the textile area [7,11,18]. Due to the number of functional groups like Si-O-Si, the material exhibits specific vibration frequencies that result in emissivity peaks, especially within the atmospheric window (8–13 µm) [19]. It is mixed with the crosslinking agent (525, Wacker Chemie AG, Germany) in a ratio of 100:3. The silicone is an addition-curing type. Silicone elastomers are reactive coating materials, allowing for chemical bonding to the substrate surface during crosslinking and ensuring a strong and durable adhesion of the coating. Properties of the materials used can be extracted from Table S1 in the Supplementary Materials.

The coating consists of two functional layers. The first layer is based on aluminum as an underlying material. Aluminum particles are directly integrated into the silicone matrix material. For the second layer, white pigments like TiO_2_ (The Chemours Company, Wilmington, DE, USA) are added to the silicone matrix. TiO_2_ particles reach, based on their refractive index, particle size and particle distribution, a high solar reflectivity, especially in the visible range [20,21,22]. The first layer with aluminum particles serves as a non-transparent layer, resulting in an opaque coating. The second layer, consisting of TiO_2_ particles integrated into silicone, acts as a solar reflective layer, increasing solar reflectivity in the visible range. The systems are stirred for 3 min at 800 rpm to achieve a uniformly distributed paste. 

### 2.4. Coating Application

The first paste is applied onto the textile substrate using a doctor blade technique and dried for 3 min at 100 °C, followed by crosslinking for 3 min at 150 °C. After that, the second functional layer is added directly onto the first cured layer using the same process. 

### 2.5. Spectral Measurements

The solar reflectivity and transmissivity in the range of 0.3–2.5 μm are obtained using an ultraviolet–visible–near-infrared (UV–Vis–NIR) spectrometer (Lambda 1050+, PerkinElmer, Shelton, CT, USA), while the infrared emissivity in the range of 2.5–20 µm is measured using a Fourier transform infrared (FTIR) spectrometer (Vertex 80, Bruker, Billerica, MA, USA). For reflectance standardization, an integrating sphere coated with highly diffuse reflective materials is employed. In the Lambda 1050+ spectrometer, a 150 mm InGaAs (Indium–Gallium–Arsenide) detector and an integrating sphere with a Spectralon^®^ inner coating are used. Before each new series of measurements, a baseline reading with T% = 100 is performed using the calibration standard Spectralon^®^ (Labsphere, North Sutton, NH, USA).

In the Vertex 80, a gold-coated integrating sphere with a DLaTgs (Deuterated L-Alanine doped Triglycine Sulfate) detector is used. Before each new series of measurements, a background measurement is taken using the gold standard without the sample. 

The measurement of the samples takes place at room temperature. For both devices, a sample size of 5 × 5 cm is used. The measurement is carried out following ASTM- E- 903:2020 [23] (Standard Test Method for Solar Absorptance, Reflectance, and Transmittance of Materials Using Integrating Spheres).

### 2.6. Outdoor Test Module

To measure the temperature differences using different background materials as well as evaluate the modeled results with real weather data, a test setup was built on the roof of the German Institutes for Textile and Fiber Research (DITF) (48°42′02.6″ N, 9°20′36.8″ E). The test setup is mounted approximately one meter above the roof surface so that the heat radiation from the ground does not have a significant impact on the measurement. By implementing a feedback-controlled heating plate system, the cooler temperature (T_s_) is kept equal to the ambient temperature (T_amb_). The temperature difference between the environment and the textile sample is kept to less than 0.2 °C throughout the entire measurement period. Influences from convection or conduction are thus further reduced. Expanded polystyrene (Styrofoam) is used as an insulation material. The Styrofoam is equipped with solar-reflective self-adhesive aluminum foil (Calorique, Düren, Germany) both on the inside and outside to reduce conduction. 

**Figure 2:**
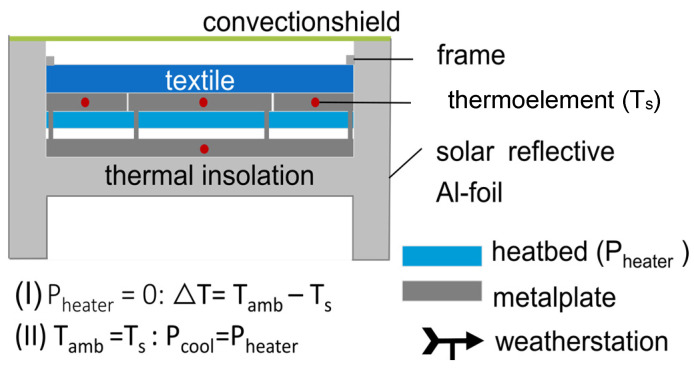
Schematic representation of the test module with a feedback-controlled heating plate system.

The schematic representation of the test module can be seen in Figure 2. A more detailed visualization is available in Figure S3 of the Supplementary Materials. 

By implementing a “Guarded-Ring” system separating the metal plate into a core and a frame plate based on the standard test method for thermal conductivity (guarded hot plate) [23], a one-dimensional heat transfer between the core and the sample is ensured, and side losses are limited [24]. The measuring area of the core and, thus, the surface of the material is 100 ± 0.5 cm^2^. 

To measure the cooling capacity in watts per square meter, self-adhesive silicone heating mats (RS Components GmbH, Frankfurt am Main, Germany) are attached below the metal plates. The heating mats are connected to the power supply unit HMP4030 3-CH (Rohde & Schwarz, Munich, Germany). The thermocouples NiCr-Ni (Ahlborn Mess- und Regelungstechnik GmbH, Holzkirchen, Germany) of type K temperature sensors are placed in the metal plate so that the measuring tip sits exactly in the middle of the respective plate. Due to the low thickness of the aluminum plate, the exact temperature of the heating plate and, thus, T_s_ can be determined. For the frame plate, two temperature sensors are used and the average value is calculated from this. Additionally, to protect the measurement from the influence of convection, a convection shield of polyethylene (LDPE) is applied horizontally on the opening of the measurement module. 

Details regarding used devices, uncertainties, and materials can be extracted from Table 1 and Table 2.

For measuring the ambient temperature, two temperature sensors are used. Firstly, a thermocouple sensor is mounted at the height of the test modules. The measuring tip is covered with a reflective foil (aluminum) to protect it from direct sunlight while ensuring an unhindered airflow. Secondly, a digital sensor for measuring humidity, temperature, and air pressure (FHAD46C41AL05, Ahlborn Mess- und Regelungstechnik GmbH) is also mounted at the height of the test modules in a climate housing (Technoline, Wildau, Germany). The climate housing, similar to a Stevenson screen, serves to shade the temperature sensor and ensure free airflow through the louver openings. The ambient temperature is provided as an average from both sensors. 

The heating power to be applied corresponds to the cooling capacity in W/m^2^ (P_cool_ = P_heater_). The meteorological data, such as humidity, wind speed, solar radiation, or air pressure, are measured by a meteorological measurement unit (Ahlborn Mess- und Regelungstechnik GmbH) and a pyranometer (Kipp & Zonen, Delft, The Netherlands). These are mounted on a weather station located approximately three meters from the test setup on the institute’s roof.

To ensure comparability between the different test modules, the variability was checked by using aluminum foil as a sample for both test setups. The temperature of the test modules was measured during the day over a period of 40 min. A slight average deviation of 0.3 °C was measured at an average direct solar radiation of <400 W/m^2^. The deviation is within the range of the measurement accuracy of the temperature sensors so that the test modules can be regarded as identical. The result can be seen in Supplementary Materials Figure S4. 

The measurement setup shows a plausible accuracy for the temperature measurement of the sample as well as that of the ambient temperature. 

### 2.7. Model for the Combined Textile-Base System

**Figure 3:**
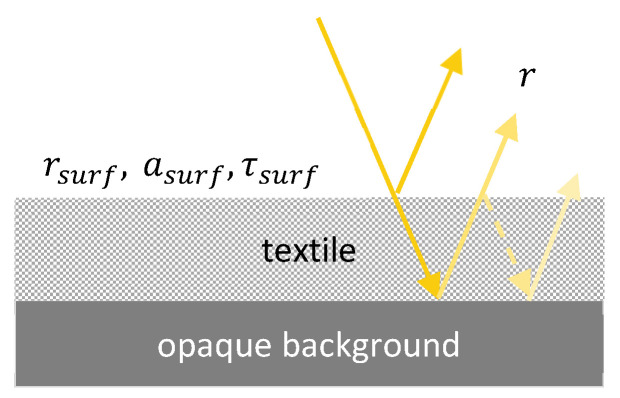
Schematic of sunlight being reflected and absorbed by a semi-transparent textile on a non-transparent base.

As illustrated in Figure 3, the solar reflectivity, transmittance, and absorptivity of the semi-transparent textile are denoted by $r_{surf},\alpha_{surf},$ and $\tau_{surf}$, respectively. And the solar reflectivity and absorptivity of the non-transparent base layer are denoted by $r_{base}$ and $\alpha_{base}$, respectively. Considering multiple reflections and absorptions of sunlight by the semi-transparent textile and the non-transparent base layer, the reflectivity of the combined textile-base system is given by [17]

(3) $$r=r_{surf}+r_{base}\frac{\tau_{surf}^{2}}{1-r_{base}r_{surf}}$$

The same rule can be used to evaluate the total spectral emissivity of the combined textile-base system. The average solar reflectivity over the solar spectrum (0.3–2.5 µm) is then given by

(4) $${\tilde{r}}_{solar}=\frac{\int_{\lambda_{0.30\;\mu m}}^{\lambda_{2.5\;\mu m}}r\left(\lambda \right)I_{solar}\left(\lambda \right)d\lambda }{\int_{0.30\;\mu m}^{2.5\;\mu m}I_{solar}\left(\lambda \right)d\lambda }$$

where λ is the electromagnetic wavelength, and $I_{solar}\left(\lambda \right)$ is the spectral solar irradiance.

Based on spectral data of the top and base material and due to the calculation of the combined solar reflectivity/MIR-emissivity, the portion of solar reflection can be classified into top contribution, base contribution, and total reflectivity/emissivity. This allows for the determination of how much each material contributes to radiative cooling and whether there is a significant influence on the base material. Based on these results, the cooling performance can be calculated and analyzed depending on different base materials. 

### 2.8. Thermal Model for Calculating Cooling Power and Temperature Differences for Textiles

**Figure 4:**
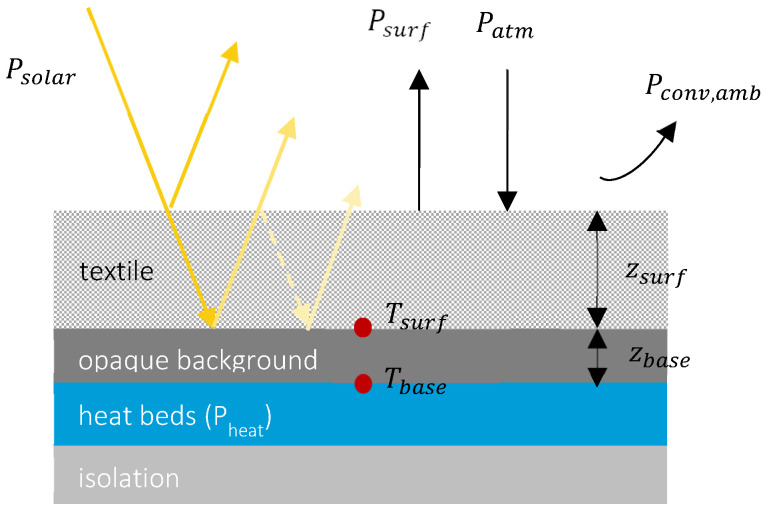
Schematic of cooling power components on the textile substrate surface.

Schematic of the textile-base system is illustrated in Figure 4, where a textile with thickness of $z_{surf}$ and temperature $T_{surf}$ is placed on top of a heated thin base with thickness of $z_{base}$ and temperature $T_{base}$. 

Components of the textile surface net cooling power are, respectively, upward longwave radiation emitted by the surface ($P_{surf\to atm,\;rad}$), downward atmospheric longwave radiation absorbed by the surface ($P_{atm\to surf,\;rad}$), incoming solar radiation absorbed by the surface ($P_{solar\to surf}$), along with convective loss between the surface and ambient temperature ($P_{conv,\;amb\to surf}$). And the heating power (optional) is denoted by $P_{heater}$. 

The net cooling of the textile surface is expressed as

(5) $$P_{surf,net\;cooling}=P_{surf\to atm,\;rad}-P_{atm\to surf,\;rad}-P_{solar\to surf}-P_{conv,amb\to surf}$$

Using the simplified but more accurate modeling approach proposed by [25,26], each component is given by

(6) $$P_{surf\to atm,\;rad}=\pi \int_{2.5}^{\infty }\varepsilon_{surf}I_{BB}\left(\lambda ,T_{surf}\right)d\lambda$$

(7) $$\begin{array}{c} P_{atm\to surf,\;rad}=\left(1-f_{cloud}\right)P_{atm\to surf,\;rad,clear}+f_{cloud}P_{atm\to surf,\;rad,cloud}\\ =\left(1-f_{cloud}\right)\pi \int_{2.5}^{\infty }\varepsilon_{surf}\varepsilon_{atm}I_{BB}\left(\lambda ,T_{amb},PW\right)d\lambda +f_{cloud}\pi \int_{2.5}^{\infty }\varepsilon_{surf}I_{BB}\left(\lambda ,T_{cloud}\right)d\lambda \end{array}$$

(8) $$P_{solar\to surf}=\left(1-{\tilde{r}}_{solar}\right)P_{sun}$$

(9) $$P_{conv,amb\to surf}=h_{surf\to amb}\left(T_{surf}-T_{amb}\right)$$

In the above equation, $\varepsilon_{surf}$ and $\varepsilon_{atm}$ are, respectively, the zenith-0° emissivity of the combined textile-base system; $P_{sun}$ is the incoming solar irradiance; $T_{amb}$ is the measurable ambient temperature just above the surface; $f_{cloud}$ is the cloud fraction; $T_{cloud}$ is the cloud temperature; *PW* is the atmospheric precipitable water; $I_{BB}$ is the blackbody spectral radiation; and $h_{surf\to amb}$ is the air convective heat transfer coefficient. 

Based on Aili et al. [27] and Martin et al. [28], it is considered that under a fully clouded sky, the clouds are closer to the Earth’s surface and can be described as a black body. With a partly clouded sky, the cloud base is higher and, therefore, colder. The parameter $f_{cloud}$ is proportional exponential to $T_{cloud}$. The emissivity of the clouds is furthermore considered to be close to one, given that clouds consist of ice crystals [29]. $T_{cloud}$ can be then calculated based on [25,28]

(10) $$T_{cloud}=\ln\left(f_{cloud}\right)\Delta T_{o}+T_{amb}$$

$\Delta T_{o}$ is a reference temperature difference and given by [25] $\Delta T_{o}=10\;℃$.

PW is calculated based on the Clausius–Clapeyron equation and Ruckstuhl et al. [30] by:

(11) $$PW\approx a\cdot RH\frac{3800\exp\left(\frac{17.63T_{amb}}{T_{amb}+243.04}\right)}{p_{amb}}-b$$

where RH and $p_{amb}$ are, respectively, the measurable relative humidity and ambient pressure, and *a* and *b* are constants that weakly depend on geographical location or altitude and are assumed by *a* = 2.1 and *b* = 0.8 [27,30]. 

The influence of the wind velocity on the wind coefficient $h_{surf\to amb}$ can be quantified for flat surfaces using the linear correlation

(12) $$h_{surf\to amb}=c\cdot V_{wind}+d$$

where *c* and *d* are constants, and $V_{wind}$ is the wind velocity. Without windshield $h_{surf\to amb}=0.53V_{wind}+5.7$, and with windshield, $0\;V_{wind}+5.7$ is considered [31]. 

During a dynamic cooling process, heat is gradually transferred from the heat source to the base layer, to the textile, and then to the sky. The transient energy balance equations associated with the base layer and the textile surface are, respectively, given as

(13) $$A_{base}P_{heat}-A_{base}\left(T_{base}-T_{surf}\right)\frac{k_{surf}}{z_{base}}=m_{base}c_{p,base}\frac{dT_{base}}{dt}$$

(14) $$A_{base}\left(T_{base}-T_{surf}\right)\frac{k_{surf}}{z_{base}}-A_{surf}P_{surf,net\;cooling}=m_{surf}c_{p,surf}\frac{dT_{surf}}{dt}$$

$A_{base}$ and $A_{surf}$ describe the surface areas of the base layer and the sample area, respectively, and can be extracted from Section 2.6. The thermal conductivity of the textile $k_{surf}$ is measured based on DIN 52,616 [24] using a heat flow measuring plate device. $m_{base}$ and $m_{surf}$ are the masses of the base and sample, while $c_{p,base}$ and $c_{p,surf}$ represent the specific heat capacities of the two materials, respectively. 

By defining the parameters such as solar radiation (W/m^2^), ambient temperature (°C), sample temperature (°C), wind velocity (m/s), relative humidity (%), and the ambient pressure (mbar), the cooling capacity can be calculated for specific weather scenarios.

## 3. Results

### 3.1. Comparability of Semi-Transparent and Non-Transparent Textile Samples on an Opaque Background

#### 3.1.1. Evaluation of the Influence of Background Materials on the Reflectance Behavior of Semi-Transparent Textiles

**Figure 5:**
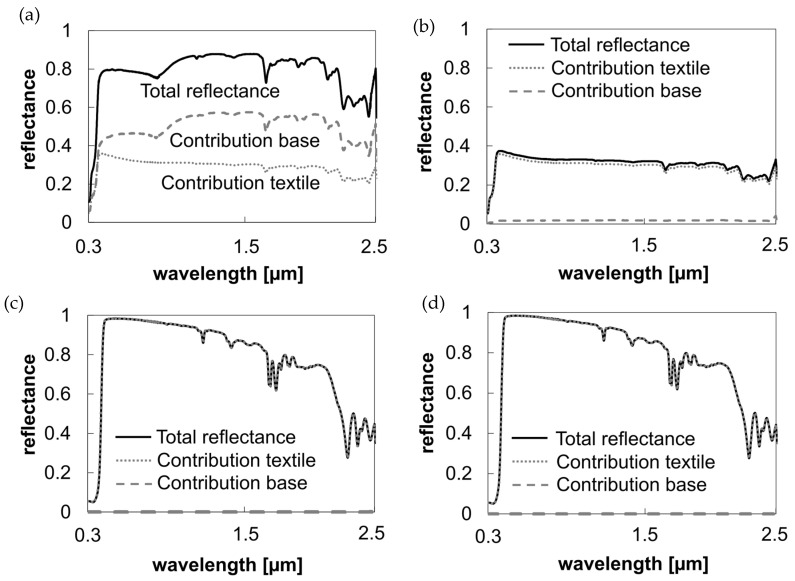
Influence of material components on the total reflection of the textile-base system. (**a**) Solar reflection: Uncoated polyester sample with aluminum foil as the background material. (**b**) Solar reflection: Uncoated polyester sample with black foil as the background material. (**c**) Solar reflection: Coated opaque textile with aluminum foil as the background material. (**d**) Solar reflection: Coated opaque textile with black foil as the background material.

The reflectance behavior of semi-transparent textiles is significantly influenced by the background material. For instance, using a highly reflective material like aluminum foil as the background enhances the overall solar reflection (see Figure 5a). An average increase of 0.5 (between 0.3 and 2.5 µm) compared to the sole reflectivity of the polyester sample is achieved. Conversely, a dark background like black foil absorbs all transmitted light, making the overall reflectance similar to the semi-transparent sample itself (see Figure 5b). 

Opaque materials, such as textiles with non-transparent cooling coatings, do not exhibit changes in spectral profiles based on the background material (see Figure 5c,d).

The coating ensures consistent cooling performance regardless of the underlying background, indicating that the reflectance and emissivity properties are dominated by the coating itself. 

#### 3.1.2. Evaluation of the Influence of Background Materials on the Emittance Behavior of Semi-Transparent Textiles

**Figure 6:**
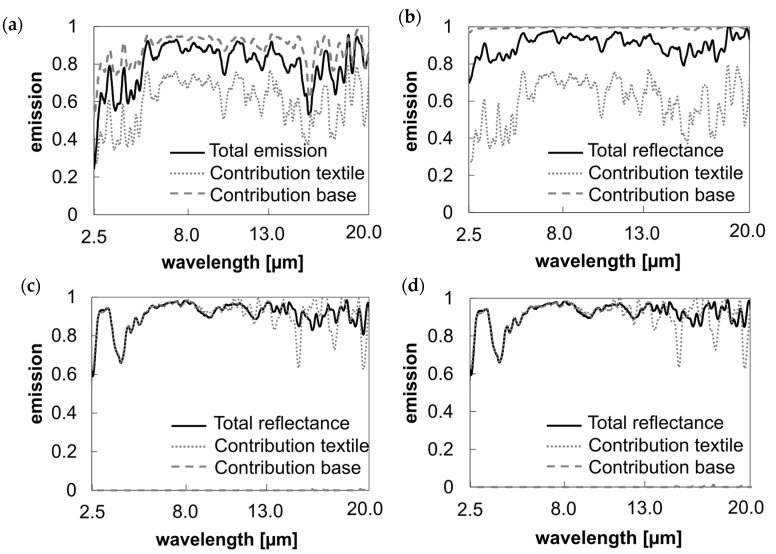
Influence of material components on the total emission of the textile-base system. (**a**) Emission/absorption MIR: Uncoated polyester sample with aluminum foil as the background material. (**b**) Emission/absorption MIR: Uncoated polyester sample with black foil as the background material. (**c**) Emission/absorption MIR: Coated opaque textile with aluminum foil as the background material. (**d**) Emission/absorption MIR: Coated opaque textile with black foil as the background material.

The same effect can be seen in the overall emissivity value in the mid-infrared (Figure 6).

Despite the fact that the emissivity of pure aluminum foil in the mid-infrared range is close to zero, an average increase in total emissivity between 8 and 13 µm of 0.18 is achieved (see Figure 6a). This is attributed to reduced mid-infrared transmittance and reflection in the combined system. 

By analyzing the spectral data of individual materials and their combinations, different samples can be tested to highlight their influence on the spectral curve and cooling capacity. This enables a comprehensive and simplified comparison of cooling performance under various conditions. 

**Figure 7:**
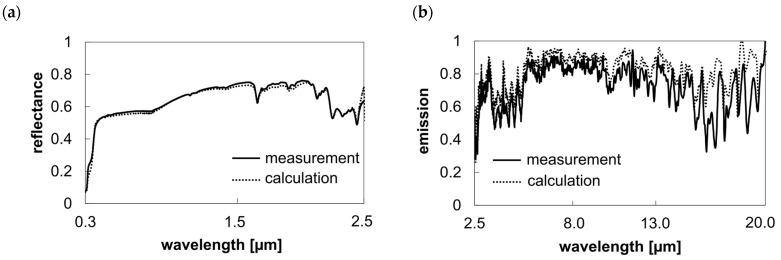
Comparison of the calculated and measured spectral curves of the combined system using the example of PES 65 g/m^2^ on an aluminum metal plate (semi-transparent textile and opaque background). (**a**) Solar reflectance; (**b**) emission in the mid-infrared.

To verify the calculated spectral response of combined systems, measurements were conducted with textiles placed directly on different background materials. The calculated and measured spectral responses in both the solar and mid-infrared ranges showed high consistency. The discrepancy in the solar range (0.3–2.5 µm) for the polyester sample on an aluminum plate was 1.4% on average (see Figure 7). 

### 3.2. Calculated Cooling Power for Different Background Materials

**Figure 8:**
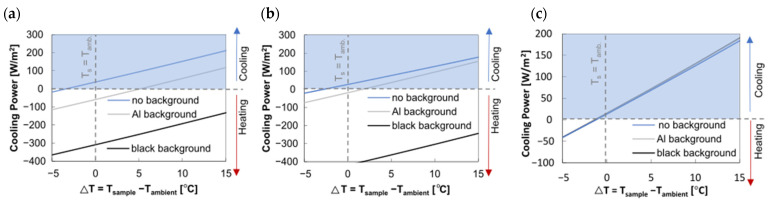
Calculated cooling power depending on different background materials. Environmental parameters: ambient temperature (Tamb): 25 °C; wind speed: 0 m/s; relative humidity: 40%; solar irradiance: 700 W/m^2^. (**a**) Uncoated semi-transparent PA6.6 sample cooling power for △T = 0: PA6.6 with no background: 37.49 W/m^2^; PA6.6–aluminum: −59.19 W/m^2^; PA6.6–black foil: −309.26 W/m^2^. (**b**) Uncoated semi-transparent PES sample cooling power for ΔT = 0: PES no background: 26.79 W/m^2^; PES–aluminum: −18.75 W/m^2^; PES–black foil: −419.62 W/m^2^. (**c**) Coated PES sample, cooling power for ΔT = 0: PES (coated) with no background: 13.03 W/m^2^; PES–aluminum (coated): 14.69 W/m^2^; PES–black foil (coated): 14.93 W/m^2^.

Using the calculated spectral curves for the textile-base system, the cooling power for semi-transparent textiles like polyester and polyamide, as well as the coated opaque textile sample, was determined. Polyamide exhibits a 7.55% lower solar transmission compared to polyester. When the solar absorption is calculated based on Kirchhoff’s law for the textile without applying any opaque background, the spectral data show lower solar absorption due to its moderate solar reflection and transmission, resulting in higher cooling power (Figure 8a). This scenario does not correspond to any practical application. Once an opaque background is considered, the cooling power decreases depending on the solar reflectance and transmittance of the sample material. 

When aluminum is used as the background material, reflecting strongly in the solar range, the cooling power in combination with the uncoated semi-transparent textile samples at thermal equilibrium (T_amb_ = T_s_) is −18.75 W/m^2^ for polyester and −59.19 W/m^2^ for polyamide. Due to the lower solar transmissivity of polyamide, the influence of the underlying background material is reduced. This results in lower cooling power with the aluminum background but also higher cooling power with a black foil sample compared to the polyester sample. Generally, the cooling power decreases significantly with a black foil background for both semi-transparent samples due to increased solar absorption (Figure 8a,b).

For the coated opaque sample, the background material does not affect cooling power, maintaining constant performance across different substrates (Figure 8c). 

### 3.3. Validation of the Thermal Model 

The thermal model was validated using real weather conditions and measurement data from the measurement setup on the rooftop. By using a loop system, the cooling power and temperature decrease can be calculated for multiple hours or days. 

Input parameters included measured data such as solar intensity, wind speed, relative humidity, ambient pressure, sample and ambient temperature, and spectral data. Calculated parameters included atmospheric emission and literature-based coefficients like $h_{air}$. The accuracy of the thermal model thus depends on the accuracy of the individual input parameters. 

Precipitable water (*PW*) significantly influences the effective atmospheric emission, especially between 8 and 13 µm, which corresponds to the atmospheric window. *PW* cannot be measured directly by the weather station but is calculated using the function and measurement data of relative humidity and ambient temperature, as described in Section 2.8. PW was approximated using minimum values typical for Central Europe [32]. The minimum value for *PW* is determined over the measurement period with an uncertainty of ±5. 

The calculations result in a slight overestimation of solar absorption by up to 2%. This could be due to discrepancies in the zenith angle measurements. Spectral data are measured at a specific angle of 8°. Recent research showed that for silicone materials, for example, the reflectivity increased by up to 30% at higher incident angles (60–80°) of solar light [33], resulting in lower absorption values. Thus, potentially higher solar reflectance of the textile samples at higher incident angles of solar light may result in a lower overall solar absorption level. Solar absorption is adjusted by −0.02.

The heat transfer coefficient, $h_{surf\to amb}$, is derived from calculated and simulated values in the literature [27,31]. Considering the maximum and minimum values for $h_{surf\to amb}$ based on variations in the experimental setup and simplifications, an uncertainty of ±5 is given. This is due to considerations of influences from the experimental setup, environmental conditions like wind speed, ambient temperature and humidity, and radiative conditions. 

#### 3.3.1. Temperature Measurement

Considering the individual influencing factors, the temperature curves and the cooling capacity values can be simulated and specified as a function of specific weather data. The input parameters are used as described, measured, calculated, or specified in the methodology. To validate the cooling model, we measured the temperature under real weather conditions for both the uncoated and coated samples using the metal plate and black foil as the underlying background material. The samples were positioned on the measurement device, as described in Section 2.6. 

**Figure 9:**
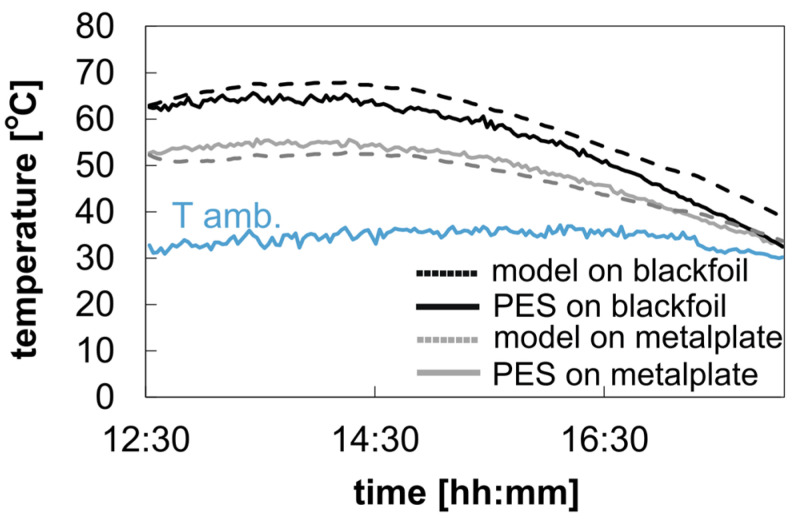
Measurement data from 10 September 2023: PW: 19.7–5 mm; solar absorption with black background: 0.72–0.02; with aluminum metal plate background: 0.39–0.02; *hsurf→amb*: 5.7 + 5 W/m^2^∙K; average solar radiation: 752.9 W/m^2^; average wind velocity: 0.9 m/s.

**Figure 10:**
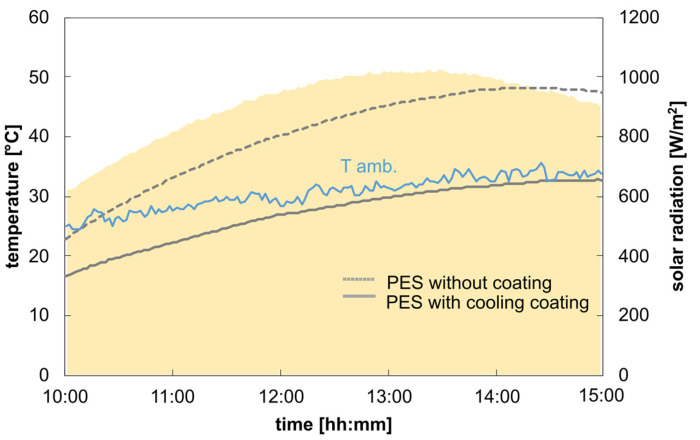
Measurement data from 8 September 2023: average solar radiation: 911.5 W/m^2^; average wind velocity: 0.6 m/s.

Figure 9 shows the temperature results for the uncoated polyester sample placed on the two different background materials. The temperature data reveal an average temperature difference of 6.9 °C between a metal plate and black foil for the uncoated polyester samples, showing the impact of the different background materials on the temperature curve for a semi-transparent textile. The temperature increase above the ambient temperature is due to the higher solar absorption value of the combined system (uncoated polyester on a metal plate or a black foil). The average solar absorption value of the two samples is about 0.41 and 0.71 for the metal plate and the black foil as underlying background materials, respectively. This also explains the high temperature difference between the two underlying background materials and their influence on the overall temperature measurement. The calculated and measured temperature curves aligned closely, with deviations of 1.5 °C and 3.7 °C for metal plate and black foil backgrounds, respectively. Figure 10 shows the impact of the coating application on the temperature results compared to the uncoated sample. Both samples are placed on the metal plate used as the background material. 

The coated opaque sample shows a temperature reduction below ambient temperature, achieving a cooling of 3.3 °C below ambient temperature during the measurement period. The cooling value is achieved due to a much lower overall solar absorptivity of about 0.12 compared to the uncoated textile sample.

#### 3.3.2. Cooling Power Measurement

In another measurement, we evaluated the cooling power for the coated opaque cooling sample, which was also placed directly on two different background materials (a metal plate and the metal plate covered with black foil). The cooling power was measured and is given by P_heater_ = P_cool_.

**Figure 11:**
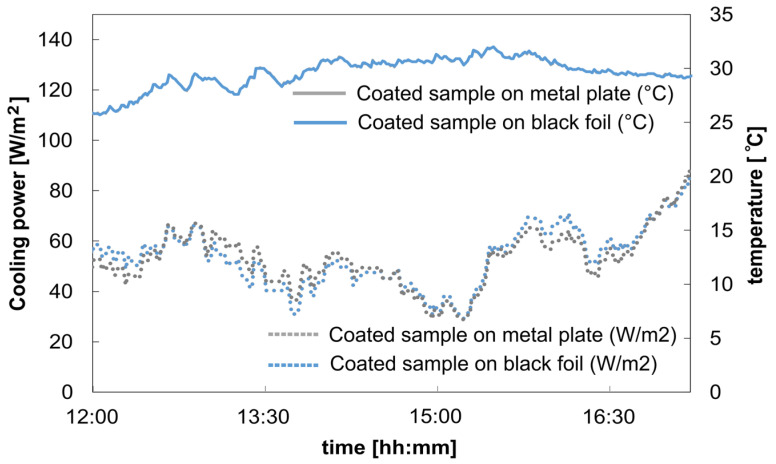
Measurement data from 13 October 2023 coated opaque PES sample on metal plate and metal plate with covered black foil: average solar radiation: 728.84 W/m^2^; average wind velocity: 1.68 m/s.

The measured cooling power for both samples is consistent on the same level, and the deviation of a maximum of 0.1 °C falls within the range of the thermocouple uncertainty. The cooling power remains closely the same, with 57.7 ± 10 W/m^2^ for the sample on the metal plate and 58.2 ± 10 W/m^2^ for the sample on the black foil. This indicates that the underlying background has no significant influence on the coated sample (Figure 11).

**Figure 12:**
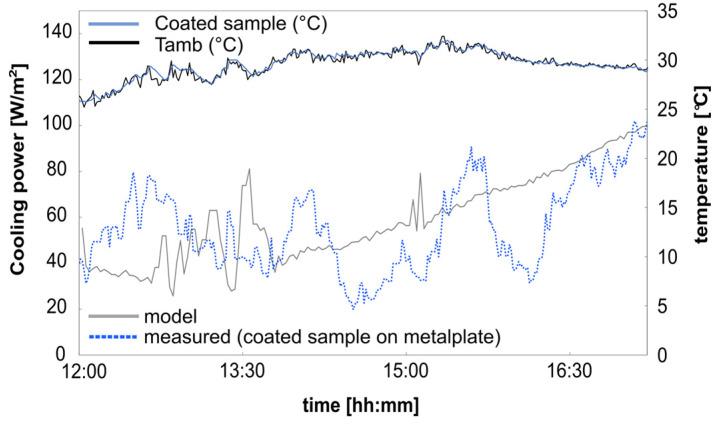
Measurement data from 13 October 2023: PW: 6.11–5 mm; solar absorption with aluminum metal plate background: 0.12–0.02; *hsurf → amb*: 5.7 + 5 W/m^2^∙K; average solar radiation: 728.84 W/m^2^; average wind velocity: 1.68 m/s.

During the test period, the precipitable water value was around 6.11 mm (see Figure 12), which was rather low. This low precipitable water value results in higher transparency, especially in the atmospheric window between 8 and 13 µm, which can lead additionally to a higher cooling value. Figure 12 illustrates the calculated cooling power of the coated sample on the metal plate compared to the measured values over midday. Due to the intermittent energy supply from the heating mats, the measured data exhibit some noise. The heating power is regulated based on temperature differences between the sample temperature (T_s_) and the ambient temperature (T_amb_). Heating stops when T_s_ equals T_amb_ and activates when T_s_ < T_amb_. This control mechanism causes more noise due to more drastic changes in wind and fluctuating solar radiation, which is more pronounced during the day than at night. Additionally, on the day of the measurement, there were sporadic appearances of clouds, which directly impacted solar radiation and resulted in bigger peaks in the measured data.

The calculated cooling power, averaging 59.5 ± 10 W/m^2^ over the test period, closely corresponds to the measured cooling power of 57.7 ± 10 W/m^2^.

This allows the model to be used to predict the cooling performance of textile samples with different background materials and simulate them under various weather scenarios.

## 4. Discussion

The findings underscore the importance of considering comparable underlying background materials, particularly in the context of samples with higher solar transparency values. 

Semi-transparent textiles play a crucial role in validating the cooling performance of functionalized textiles or in clothing applications where permeability and, thus, solar transparency are common. When reflectivity and transmissivity are measured using spectrometers, the results reflect the intrinsic properties of the textile sample. However, applying Kirchhoff’s law based on these spectral data may suggest good cooling performance for semi-transparent samples but does not accurately represent application scenarios with opaque background materials. 

Typically, textiles are placed on an opaque background like a metal plate or silicone mat, similar to artificial human skin in measurement devices. This is also true for real application scenarios like car covers, covers of roofs, or clothing materials. Therefore, understanding and accounting for the impact of the underlying background is essential for accurately predicting and optimizing the cooling performance of textile materials. 

An opaque background can lead to an increase or decrease in cooling power depending on the solar reflection and transmittance of the sample material. The results show that the more transparent the sample, the more influence the underlying material has on the overall temperature reduction and cooling power. 

Additionally, this study emphasizes the importance of high solar reflectivity and low solar transparency to achieve cooling independent of the underlying background material. For coated opaque samples, the background material does not influence the cooling power, as it remains constant across different base systems due to the coating’s low transparency and high opacity, especially in the solar range.

For further material design approaches in the textile area, it is, therefore, important to also take solar transparency into account by either applying a coating with a certain thickness or adapting the design by using materials or particles like metals that can reduce the overall transmissivity.

By considering and measuring various background materials, the simulation compares transparent, semi-transparent, and opaque textiles under identical conditions, accurately characterizing the influence of the textiles’ transparency values on cooling performance. The prediction model has been successfully verified using the measured temperature profiles. The measured spectral curves enable statements about the cooling performance of different textile materials, textile-base systems, and weather conditions. 

This model facilitates the validation of cooling performance for various textile materials across different application scenarios and background materials, eliminating the need for costly test setups. By using the model along with the spectral data for the underlying background provided in the Excel sheet “Spectral data Background Materials”, the cooling capacity of textile materials can be accurately compared and validated. Thus, this process only requires the measurement of the textile’s spectral data. Moreover, the model enables testing under various environmental conditions by changing the input parameters of solar radiation, temperature, or wind velocity.

In summary, accurately predicting and optimizing textile cooling performance demands a comprehensive understanding of the impact of underlying background materials.

## Figures and Tables

**Table 1:** Devices used and their uncertainty for the measurement setup.

Devices	Model	Uncertainty
Thermoelement NiCr-Ni	Typ K	±1.5 K
Digital sensor for humidity, temperature, and air pressure	FHAD46C41AL05	±0.2 K
Data logger	ALMEMO^®^ 710	-
Meteorological sensor for wind	FMD760	±0.3 m/s
Meteorological sensor for humidity	FMD760	±2%
Meteorological sensor for air pressure	FMD760	±0.5 hPa
Global radiation sensor for UVA, VIS, and IR radiation	FLA613T1B11	<10%
Pyranometer	CM 11	<10 W/m^2^

**Table 2:** Materials used and their thickness in the measurement setup.

Material	Thickness
Insulation (Styrofoam)	10 ± 0.5 cm
Solar reflective aluminum foil (Calorique, Germany)	30 ± 5 µm
Metal plate (aluminum)	0.6 ± 0.05 cm
Silicone heating mats (RS Components GmbH, Germany)	1.4 mm
Wind cover—polyethylene (LDPE)	10 ± 5 µm

## Data Availability

Data are contained within the article and the Supplementary Materials.

## Supplementary Materials

The following supporting information can be downloaded at https://www.mdpi.com/article/10.3390/polym16162264/s1. Figure S1: Spectral curves of the two semi-transparent uncoated textile materials; Figure S2: Spectral curves of the opaque background materials; Figure S3: Assembly of the test modules for measuring cooling performance in Celsius and W/m^2^ using a feedback-controlled heating plate system; Figure S4: Comparison of the test modules; Table S1: Properties and manufacturer’s specifications of the materials used.
