# Supplementary material for: The Influence of Background Materials on the Radiative Cooling Performance of Semi-Transparent and Opaque Textiles: A Theoretical and Experimental Analysis
Source: Polymers (Basel). 2024 Aug 9;16(16):2264. doi: 10.3390/polym16162264 (PMC11360624; doi:10.3390/polym16162264)
Supplement: Supplementary file 1 [file polymers-16-02264-s001.zip › polymers-3091545-supplementary.pdf]

Supplementary Materials

Figure S1 describes the spectral curve of the uncoated textile materials of PA6.6 and PES. PA6.6, with a higher area per weight, achieves nearly 20% lower solar transmissivity compared to PES.
In the mid-infrared range, the average percentage deviation between 2.5–20 µm from PA6.6 to PES is 32.2% providing two mainly different substrate textile materials based on the spectral curve.

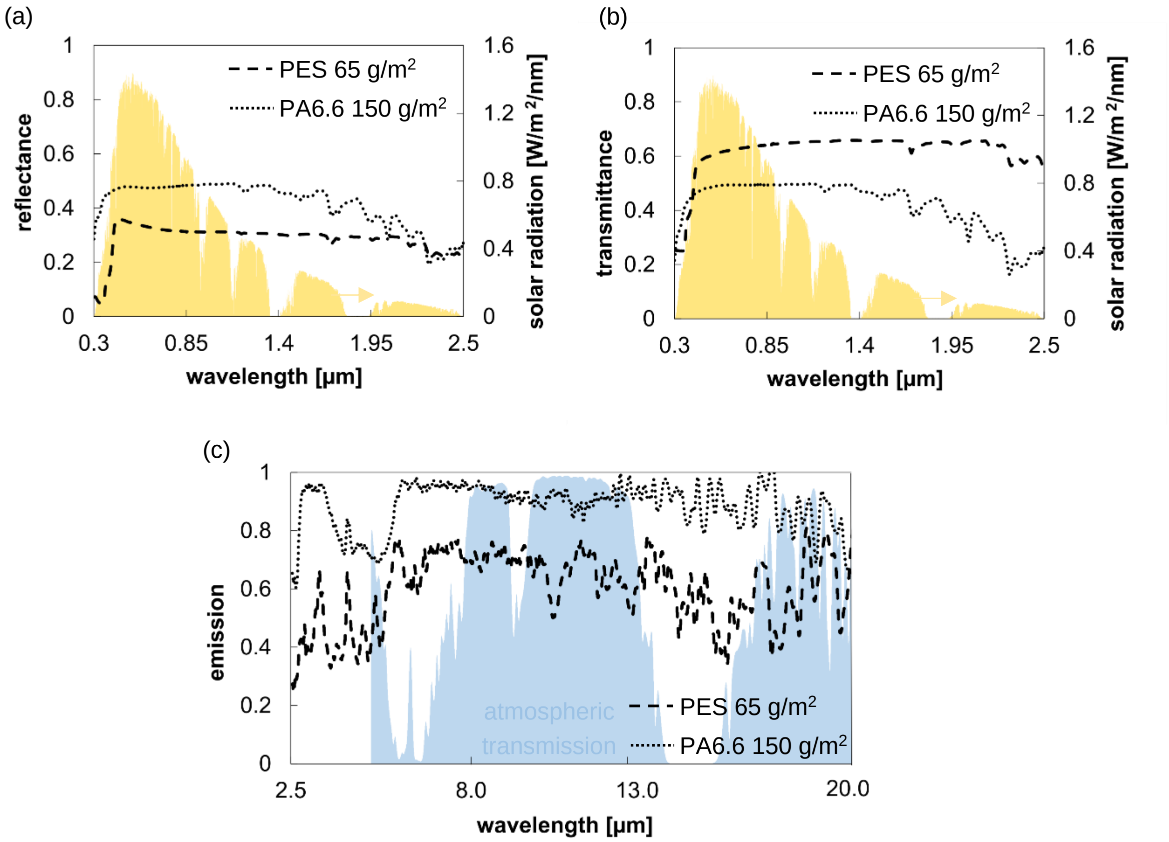

Figure S1. Spectral curves of the two semitransparent uncoated textile materials. (a) Spectral reflectance in the solar range; (b) Spectral transmittance in the solar range; (c) Spectral emission in the mid infrared range.

Figure S2 describes the spectral curves of the background materials. The black foil exhibits the lowest solar reflectance compared to the aluminum foil and the aluminum metal plate, while also showing the highest mid-infrared emission value. In contrast, aluminum foil demonstrates the highest solar reflectivity with an average level in the solar range of >90 % and the lowest emission value in the mid-infrared. The aluminum metal plate, due to higher scattering factors, falls in between these two extremes.

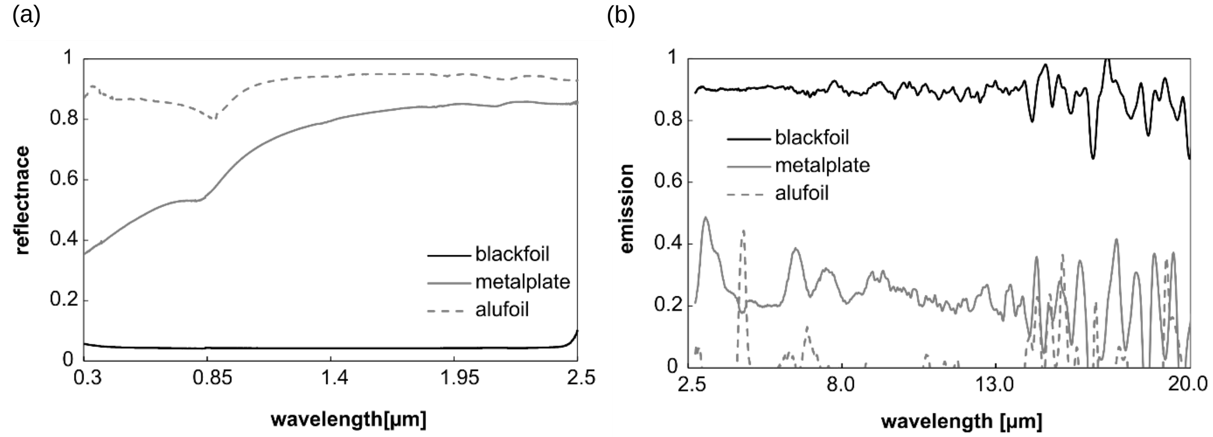

Figure S2. Spectral curves of the opaque background materials. (a) Spectral reflectance in the solar range; (b) Spectral emission in the mid infrared range.

Table S1. Properties and manufacturer's specifications of the materials used

Material	Density [g/cm³]	Optical Properties	Thickness	Viscosity (1 s⁻¹) [mPa·s]	Manufacturer Identification
ELASTOSIL® LR 6250 F TRANSPARENT	1.07	highly transparent	-	100000	Wacker Chemie AG
ELASTOSIL® Crosslinker 525	0.94–0.98	clear, colorless	-	300–500	Wacker Chemie AG
LDPE-foil	-	transparent	10±5 μm	-	GUG GROSSHANDEL
Aluminium foil	-	polished	30±5 μm	-	Calorique

Figure S3 provides a detailed overview of the test setup designed on the rooftop of DITF for measuring the temperature and cooling power of the samples.
The sample is tensioned between the inner side of the insulation box and a tension frame, so that it lies flat on the aluminium metal plate achieving good conductivity between the textile and the metal plate. The distance between the frame and core plate is 5±0.5 mm. The frame plate has an area of 456±0.5 cm². A lower metal plate, which is inserted with a distance of 5±0.5 mm to the core and frame plate, serves as a support for the upper plates. The distance between the convection shield and the sample surface is 15±0.5 mm.

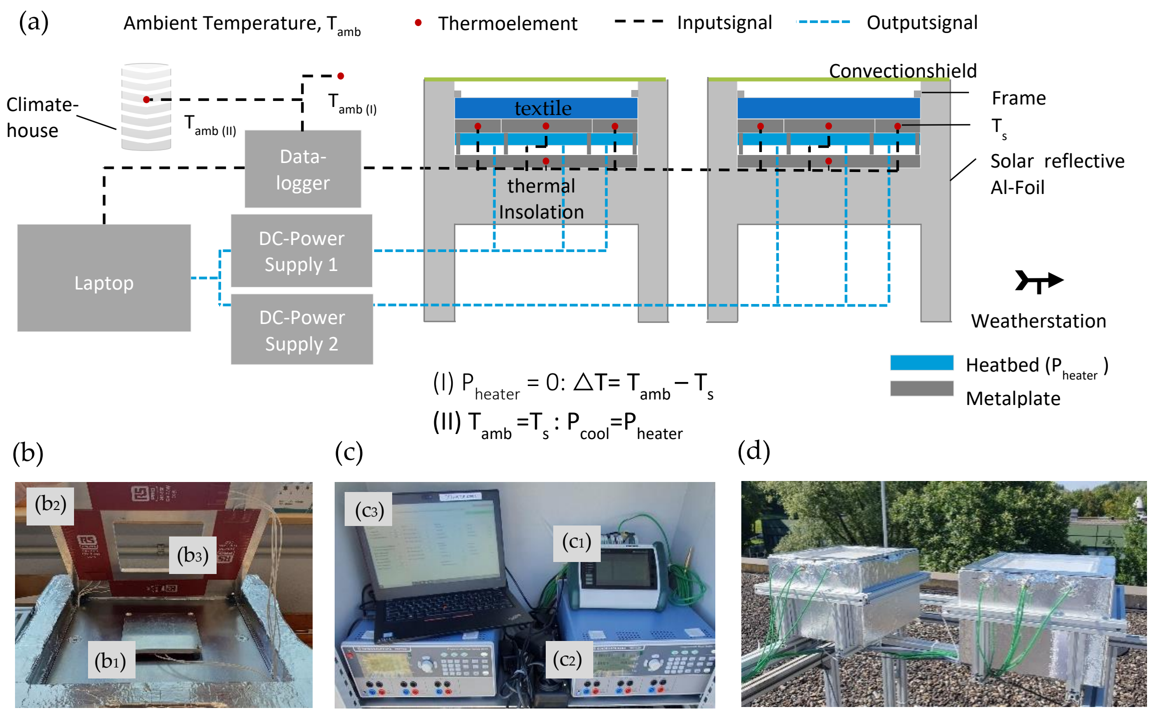

Figure S3. Assembly of the test modules for measuring cooling performance in Celsius and W/m² using a feedback-controlled heating plate system. (a) Schematic representation of the cross-sectional view of the test modules, showing the system components, data acquisition, control system, and the position of individual components and thermocouples. (b) Photograph of the "Guarded-Ring" heating plate system, (b1) Core plate, (b2) Frame plate, (b3) Heatbeds. (c) Photograph of the data acquisition, (c1) Data logger, (c2) DC power supplies (1 & 2) for each test module, (c3) Data acquisition and control of heating power; (d) Photograph of the test modules on the institute's roof.

Figure S4(a) shows temperature measurements of the two setups, indicating stable temperatures in the core metal plate and in the frame plate over a 40-minute period using aluminum foil as the sample, confirming their comparability.
Figure S4(b) demonstrates consistent temperatures in both setups for over 24 hours, further confirming their comparability over a longer duration.

Since aluminum foil used in the sample has very low emission values near zero, its temperature naturally matches ambient temperatures during nighttime when solar influence is absent. This alignment verifies that measured ambient temperatures accurately reflect those of the sample, ensuring reliable data. Any significant deviation would imply errors in temperature measurement setups or ambient temperature readings, which are not evident in these measurements.

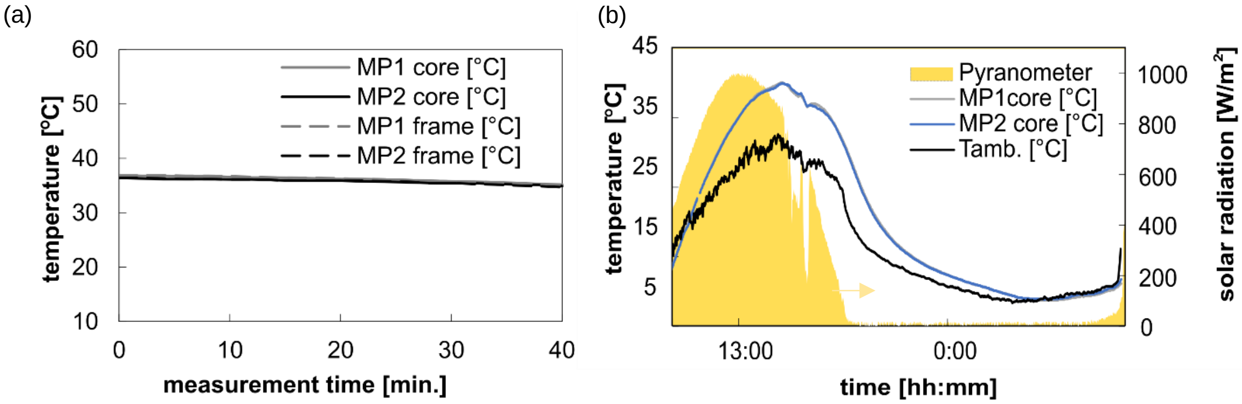

Figure S4. Comparison of the test modules. Temperature measurement over a period of 40 minutes with identical sample usage (aluminium foil). (a) Comparability of the test modules with heating plate system on 27.09.2023, 17:05 – 17:45, average solar radiation intensity: 350.8 W/m², frame and core temperatures remain at a constant level, the standard deviation is 0.3 °C; (b) Comparison of core temperature for aluminium foil on the test modules with heating plate system – the temperature of both modules remain constant at the same level for a period of up to 24 hours, the night temperature corresponds to the measured ambient temperature.
